# BML-111 Protected LPS/D-GalN-Induced Acute Liver Injury in Rats

**DOI:** 10.3390/ijms17071114

**Published:** 2016-07-13

**Authors:** Dan Yan, Hai-Ling Liu, Zhong-Jian Yu, Yong-Hong Huang, Dian Gao, Hua Hao, Shou-Sheng Liao, Fang-Yun Xu, Xiao-Yan Zhou

**Affiliations:** 1 Department of Pharmacology, Medical College of Nanchang University, Nanchang 330006, China; yandan177@sina.com (D.Y.); yuzhongjian0701@yahoo.com (Z.-J.Y.); 2 Department of Pharmacy, Jiangxi Province Cancer Hospital, Nanchang 330006, China; 3 Department of Pathophysiology, Medical College of Nanchang University, Nanchang 330006, China; liuhailing2016@sina.com (H.-L.L.); yhhuang0710@ncu.edu.cn (Y.-H.H.); scbxu999@sina.com (F.-Y.X.); 4 Department of Science and Education, Jiangxi Province Cancer Hospital, Nanchang 330006, China; 5 Jiangxi Province Key Laboratory of Tumor Etiology and Molecular Pathology, Nanchang 330006, China; 6 Department of Human Parasitology, Medical College of Nanchang University, Nanchang 330006, China; gaodian@ncu.edu.cn; 7 Department of Pathology, Second Affiliated Hospital of Nanchang University, Nanchang 330006, China; haohua410@126.com (H.H.); liaoshousheng@sina.com (S.-S.L.)

**Keywords:** lipoxins, liver injury, antioxdation, inflammation

## Abstract

Lipoxins (LXs) display unique pro-resolving and anti-inflammatory functions in a variety of inflammatory conditions. The present study was undertaken to investigate the effects of BML-111 (5(*S*),6(*R*),7-trihydroxyheptanoic acid methyl ester), the agonist of lipoxin A_4_ receptor, in a model of Lipopolysaccharides (LPS) and d-Galactosamine (d-GalN) induced acute liver injury, and to explore the mechanisms. Histopathological analyses were carried out to quantify liver injury degree. The activities of myeloperoxidase (MPO) were examined to evaluate the levels of neutrophil infiltration. The activities of aspartate aminotransferase (AST) and alanine aminotransferase (ALT) in serum were detected to evaluate the functions of the liver. The amounts of tumor necrosis factor-α (TNF-α), interleukin-10 (IL-10), and interleukin-1β (IL-1β) were measured using enzyme-linked immunosorbent assay (ELISA), and the expression levels of transforming growth factor-β1(TGF-β1) and cyclooxygenase-2 (COX-2) were examined using Western blotting. The antioxidant capacity, the activities of inducible nitric oxide synthase (iNOS), the contents of malondialdehyde (MDA) and nitric oxide (NO) were analyzed with the kits via biochemical analysis. We established the model of acute liver injury with lipopolysaccharide and d-Galactosamine (LPS/d-GalN): (1) histopathological results and MPO activities, with the activities of AST and ALT in serum, consistently demonstrated LPS and d-GalN challenge could cause severe liver damage, but BML-111 could prevent pathological changes, inhibit neutrophil infiltration, and improve the hepatic function; (2) LPS/d-GalN increased TNF-α, IL-1β, COX-2, and IL-10, while decreasing TGF-β1. However, BML-111 could repress LPS/d-GalN -induced TNF-α, IL-1β and COX-2, meanwhile increasing the expression levels of TGF-β1 and IL-10; (3) LPS/d-GalN inhibited the activities of superoxide dismutase (SOD), catalase (CAT), total antioxidant capacity (T-AOC), and hydroxyl radical-scavenging ability, simultaneously increasing the levels of MDA and NO, so also the activity of iNOS. Otherwise, BML-111 could reverse all the phenomena. In a word, BML-111 played a protective role in acute liver injury induced by LPS and d-GalN in rats, through improving antioxidant capacity and regulating the balance of inflammatory cytokines.

## 1. Introduction

Lipoxins (LXs) are biologically active productions of arachidonic acid, and are typically generated via cell interactions, which is called the transcellular synthesis pathway [1,2]. LXs exert potent pro-resolving and anti-inflammatory functions primarily [3,4]. The rapid inactivation kinetics of LXs have complicated their analyses in vivo, and prompted the synthesis of biologically active LXs analogues with enhanced chemical stability [5]. BML-111 (5(*S*),6(*R*),7-trihydroxyheptanoic acid methyl ester), the agonist of lipoxin A_4_ receptor (ALX), is the most widely used LX analogue, and was initially shown in 1991 to be equiactive with lipoxin A_4_ (LXA_4_) with respect to the inhibition of LTB4-induced neutrophil migration [6]. Subsequent studies have demonstrated potent anti-inflammatory properties of BML-111 in a variety of inflammatory conditions, including acute lung injury [7,8,9,10,11], arthritis [12], sepsis [13], ischemia/reperfusion injury [14], and ischemic stroke [15]. We and others have reported that BML-111 also protected against hepatic fibrosis [16] and hepatocellular carcinoma [17,18,19]. However, the effects of BML-111 on acute liver injury are still unclear. The aim of this study was to observe the effects of BML-111 on Lipopolysaccharides and d-Galactosamine (LPS/d-GalN) induced liver injury and discuss the mechanisms.

## 2. Results

### 2.1. BML-111 Protected Acute Liver Injury Induced by LPS and d-GalN

**Figure 1:**
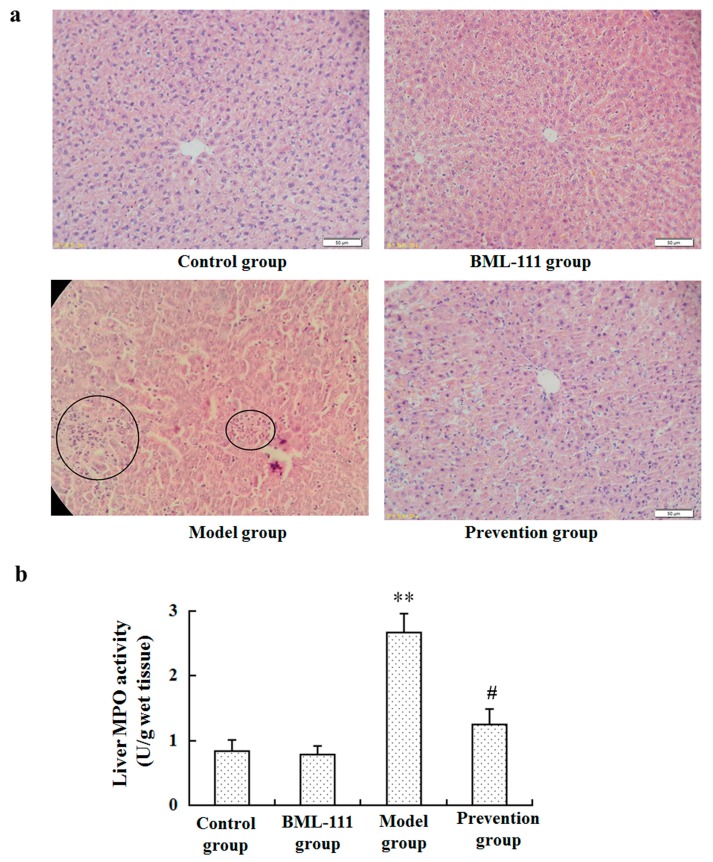
Effect of BML-111 on Lipopolysaccharides and d-Galactosamine (LPS/d-GalN) induced acute liver injury. (**a**) Haematoxylin and eosin (H&E) staining of livers (original magnification, ×200); (**b**) leukocyte infiltration was evaluated using an myeloperoxidase (MPO) kit according to the manufacturer’s protocols. ** *p* < 0.01 compared with the control group, ^#^ *p* < 0.05 compared with the model group (*n* = 6).

Histological examination in the model group identified hepatocyte necrosis with an influx of inflammatory cells, which was much less evident in the prevention group (Figure 1a). In addition, an myeloperoxidase (MPO) assay demonstrated that leukocyte infiltration dramatically declined in the prevention group compared with the rats of the model group (*p* < 0.05, Figure 1b).

### 2.2. BML-111 Repressed the Activities of ALT and AST Induced by LPS and d-GalN

**Figure 2:**
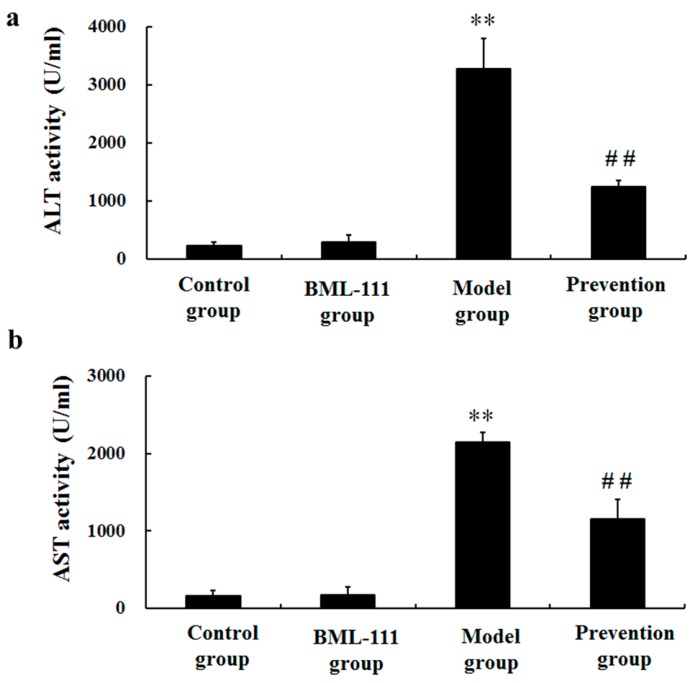
BML-111 repressed the activities of (**a**) ALT and (**b**) AST induced by LPS and d-GalN. Aspartate aminotransferase (AST) and alanine aminotransferase (ALT) were measured using kits according to the manufacturer’s protocols. ** *p* < 0.01 compared with the control group, ^##^ *p* < 0.01 compared with the model group (*n* = 6).

To observe the influences of BML-111 on liver injury induced by LPS and GalN, the activities of aspartate aminotransferase (AST) and alanine aminotransferase (ALT) in serum were assessed. LPS/d-GalN markedly increased the activities of ALT and AST. However, the activities of ALT and AST were dramatically decreased in the prevention group, compared with the model rats (*p* < 0.01, Figure 2).

### 2.3. BML-111 Inhibited The Levels of TNF-α and the Activity of Caspase 3 Induced by LPS and d-GalN

**Figure 3:**
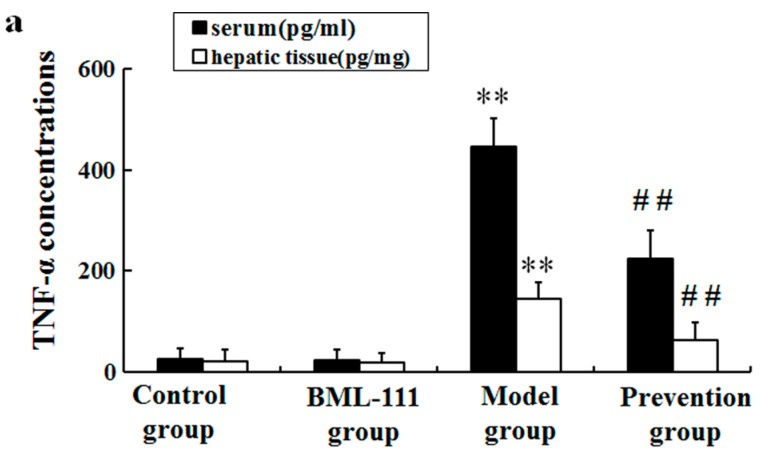

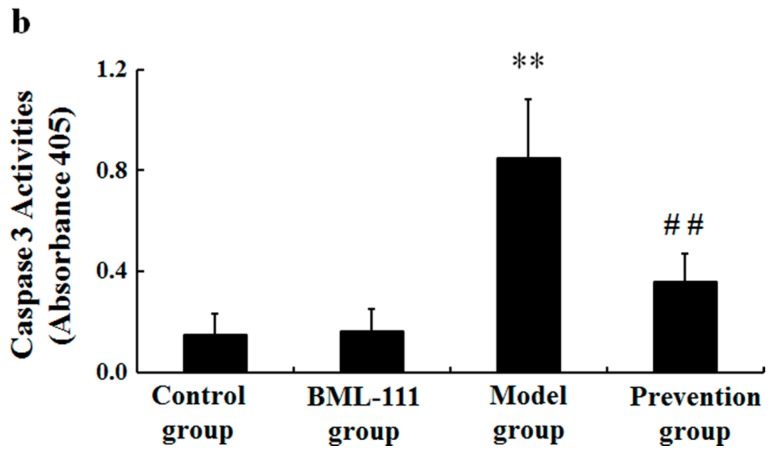
BML-111 inhibited LPS/d-GalN induced TNF-α and caspase 3 activity; (**a**) the levels of TNF-α were measured at 1.5 h after LPS/d-GalN administration in serum and hepatic tissues, using ELISA kits; (**b**) the relative activities of caspase 3 in hepatic tissue at 6 h after being challenged with LPS/d-GalN. ** *p* < 0.01 compared with the control group, ^##^ *p* < 0.01 compared with the model group (*n* = 6).

Since TNF-α is quite an important mediator of liver injury induced by LPS/d-GalN, we measured the levels of TNF-α in liver and serum at 1.5 h after administration of LPS/d-GalN. As demonstrated in Figure 3a, LPS/d-GalN markedly increased the levels of TNF-α in serum and hepatic tissues, but BML-111 significantly repressed TNF-α induced by LPS/d-GalN (Figure 3a). Furthermore, it was suggested that the activity of caspase-3 was obviously elevated at 6 h after being challenged with LPS/d-GalN. However, the increase of caspase-3 activity was also repressed in BML-111 pretreated rats (Figure 3b).

### 2.4. BML-111 Inhibited LPS/d-GalN-Induced COX-2 and IL-1β

**Figure 4:**
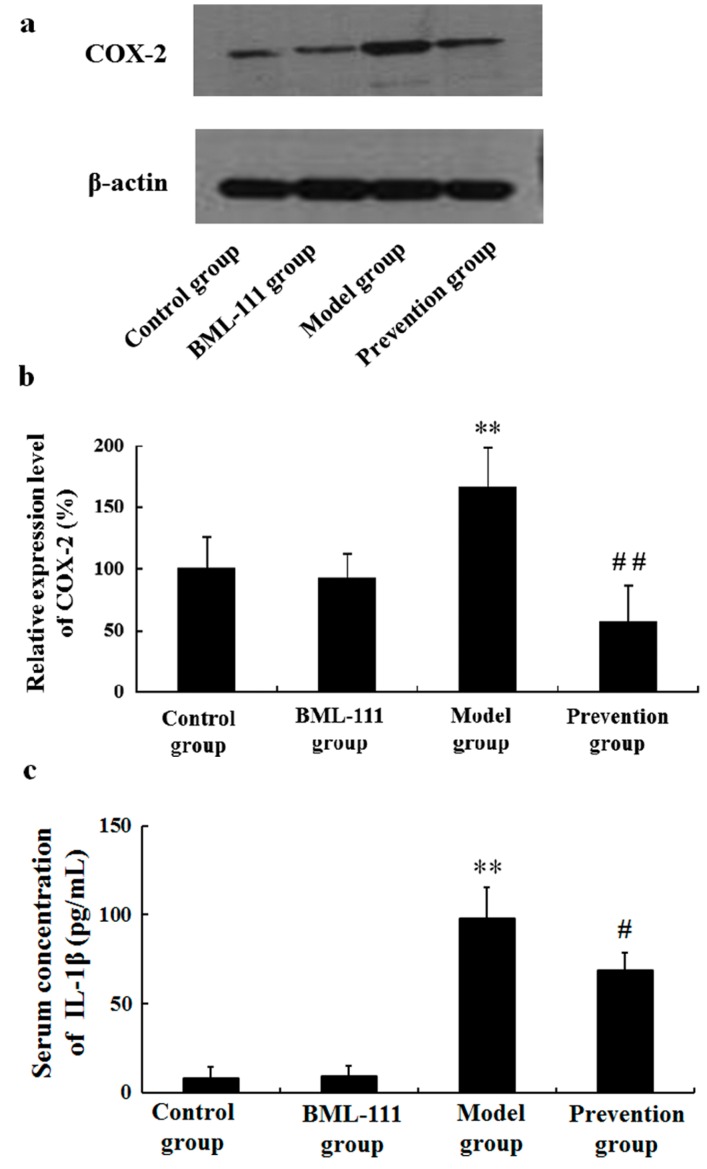
BML-111 inhibited LPS/d-GalN induced COX-2 and IL-1β. (**a**) Western blot analysis of COX-2. The COX-2 band (72 kDa) and the β-actin band (43 kDa) are indicated; (**b**) blots obtained from several experiments were analyzed using densitometry, and the densitometric values are shown as means ± SD (*n* = 3); (**c**) The levels of IL-1β in serum were measured via ELISA. ** *p* < 0.01 compared with the control group, ^#^ *p* < 0.05, ^##^ *p* < 0.01 compared with the model group (*n* = 6).

Besides TNF-α, we also observed other pro-inflammatory cytokines, including COX-2 and IL-1β. As shown in Figure 4, in the model rats, LPS/d-GalN increased the amounts of COX-2 and IL-1β, but the effects were reversed in BML-111-pretreated rats.

### 2.5. BML-111 Upregulated TGF-β and IL-10

**Figure 5:**
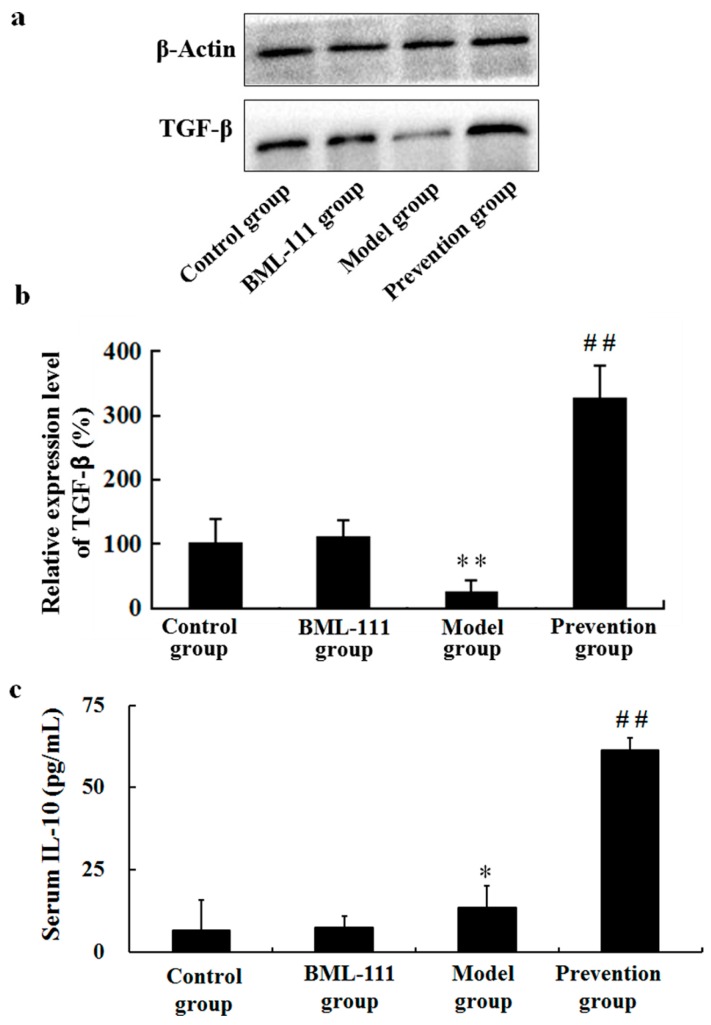
BML-111 increased the expression levels of TGF-β and IL-10. (**a**) Western blot analysis of TGF-β1. The TGF-β1 band (44 kDa) and the β-actin band (43 kDa) are indicated; (**b**) blots obtained from several experiments were analyzed using densitometry, and the densitometric values are shown as means ± SD (*n* = 3); (**c**) the levels of IL-10 in serum were measured using ELISA kits according to the manufacturer's protocols. * *p* < 0.05, ** *p* < 0.01 compared with the control group, ^##^ *p* < 0.01 compared with the model group (*n* = 6).

Results suggested decreased expression of TGF-β1 and increased expression of IL-10 in LPS/d-GalN-treated rats. However, in the prevention group, BML-111 could significantly promote the levels of TGF-β and IL-10, compared with the model rats (*p* < 0.01, Figure 5).

### 2.6. BML-111 Reduced LPS/d-GalN-Induced MDA

**Figure 6:**
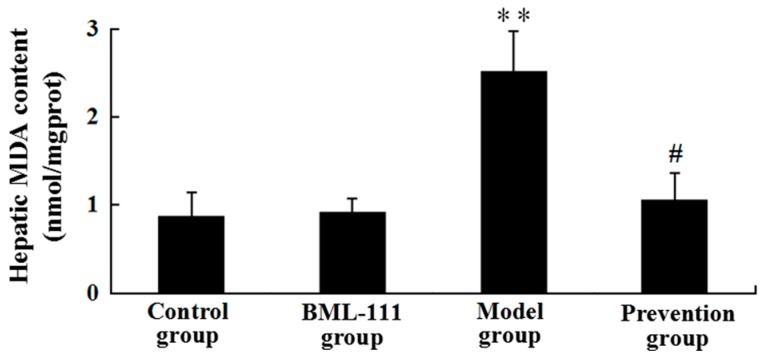
BML-111 reduced the contents of malondialdehyde (MDA). The contents of MDA were evaluated using the assay kit at 8 h after being challenged with LPS/d-GalN. ** *p* < 0.01 compared with the control group, and ^#^ *p* < 0.05 compared with the model group (*n* = 6).

MDA is generally accepted as a marker for damages induced by free radicals. Figure 6 revealed the results and comparisons of liver MDA levels. It was suggested that LPS/d-GalN increased the amount of MDA. However, compared with the model rats, the content of MDA was decreased in the prevention group (*p* < 0.05, Figure 6).

### 2.7. BML-111 Inhibited LPS/d-GalN Induced NO Production and iNOS Activity

**Figure 7:**
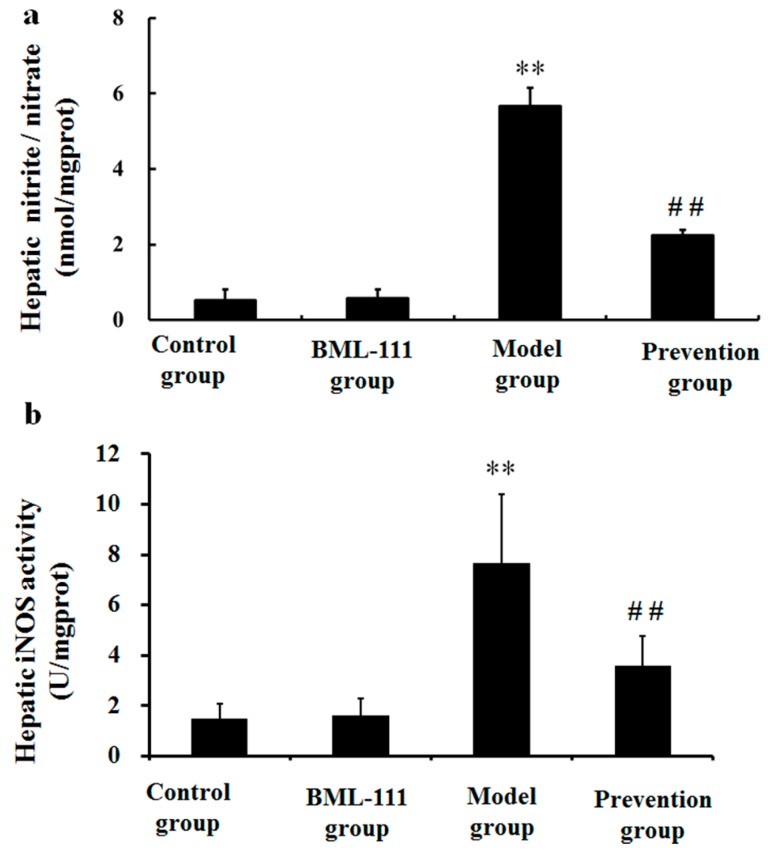
BML-111 inhibited LPS/d-GalN induced NO and iNOS activity. (**a**) The contents of NO were tested *via* assay kit at 8 h after being challenged with LPS/d-GalN; (**b**) The activities of iNOS were analyzed *via* assay kit at 8 h after LPS/d-GalN injection. ** *p* < 0.01 compared with the control group, and ^##^ *p* < 0.01 compared with the model group (*n* = 6).

We measured NO levels and iNOS activities in the supernatants of liver with the purpose of assessing the hepatic oxidative status of different groups. The data hinted that hepatic NO level was dramatically increased after an LPS/d-GalN challenge (Figure 7a). Simultaneously, the iNOS activity was also elevated markedly (Figure 7b). However, pretreatment with BML-111 significantly decreased the level of NO and the activity of iNOS (*p* < 0.01, Figure 7a,b).

### 2.8. BML-111 Improved Hepatic Antioxidant Capacity

**Figure 8:**
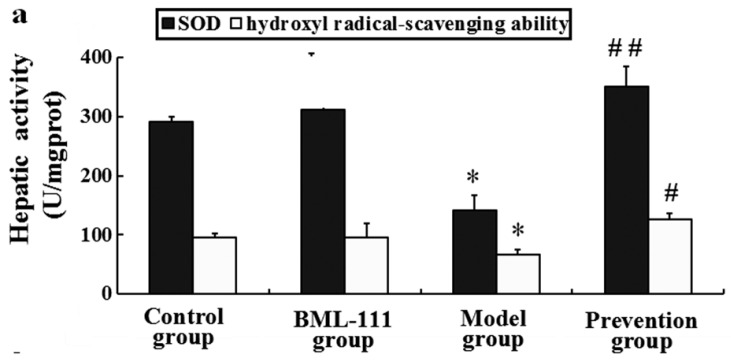

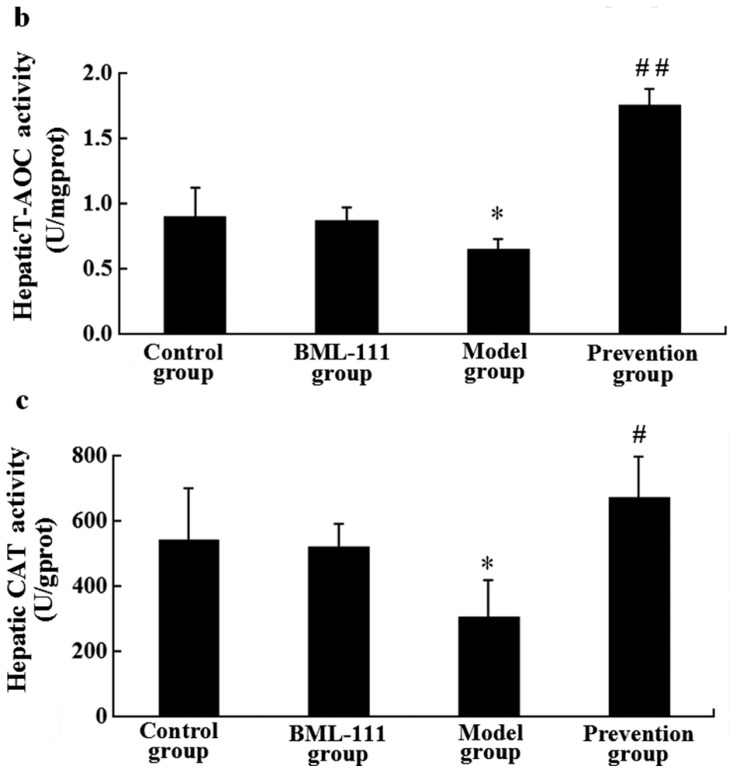
BML-111 improved hepatic antioxidant capacity. (**a**) Superoxide dismutase (SOD) and hydroxyl radical-scavenging ability were evaluated using assay kits; (**b**) Total antioxidant capacity (T-AOC) was examined by assay kit; (**c**) The activities of catalase (CAT) was tested through assay kit at 8 h after being challenged with LPS/d-GalN. * *p* < 0.05 compared with the control group, ^#^ *p* < 0.05, ^##^ *p* < 0.01 compared with model group (*n* = 6).

In the model rats, the antioxidant capacity was damaged as evinced by decreases of the activities of catalase (CAT) and superoxide dismutase (SOD), as well as the reduction of total antioxidant capacity (T-AOC) and hydroxyl radical-scavenging ability. Compared with the model rats, pre-treatment with BML-111 resulted in increased activities of SOD (*p* < 0.01, Figure 8a) and CAT (*p* < 0.05, Figure 8c), as well as T-AOC (*p* < 0.01, Figure 8b) and hydroxyl radical-scavenging potential (*p* < 0.05, Figure 8a).

## 3. Discussion

LPS and d-GalN induced liver injury is a well-known experimental model, which is very similar to fulminant hepatitis in humans [20,21]. Thus, LPS/GalN-induced model of acute liver injury was normally used to illuminate the functions of liver protecting drugs. In the present study, we found that BML-111 protected against LPS/d-GalN-induced acute liver injury. The protective effects of BML-111 were evident from improvements in leukocyte infiltration, congestion and hepatocyte necrosis, as well as improved hepatic functions reflected by decreased activities of the enzymatic biomarkers ALT and AST (Figure 1 and Figure 2). These results all adequately indicated the protective effects of BML-111 against LPS/GalN-induced liver injury in rats. Consistent with our findings, Zhang et al. and El-Agamy et al. have reported BML-111 improved carbon tetrachloride and acetaminophen induced acute liver injury in mice [22,23].

It has been reported that many factors, such as inflammation, oxidative stress, apoptosis, and immune responses, etc., could lead to acute liver injury. In this paper, we just focused on inflammation and oxidative stress. LPS works through activating Kupffer and inflammatory cells to secrete multifarious inflammatory cytokines, such as TNF-α, IL-1β, COX-2, etc. [24,25]. Among them, TNF-α is the most important pleiotropic cytokine, which can trigger an inflammatory cascade to induce other cytokines, such as IL-1β, IL-6, NO and cell adhesion molecules, etc. [26,27]. TNF-α binds to its receptor on the membrane of hepatocyte, thus activating caspase-3, and eventually inducing apoptosis [28]. In this study, LPS/d-GalN significantly increased the levels of TNF-α in serum and hepatic tissue, and the activity of caspase-3 in liver. However, BML-111 suppressed LPS/d-GalN-induced TNF-α, and diminished caspase-3 activity (Figure 3). In fact, the inflammatory process is strictly controlled by not only pro-inflammatory mediators, but also anti-inflammatory mediators. IL-10 is known as an important endogenous anti-inflammatory cytokine, mainly produced by Th cells, which can inhibit the production of TNF-α, IL-1β, IL-6 and IL-8, etc., thereby reducing inflammation and strengthening the protection of liver cells. In addition, IL-10 can reduce liver injury through inhibiting the infiltration and activation of neutrophils, preventing neutrophil adhesion to sinusoidal endothelial cell. In this study, the data demonstrated that LPS/d-GalN increased the levels of pro-inflammatory mediators, and decreased the amounts of anti-inflammatory cytokines; however, BML-111 could reserve all the effects significantly (Figure 3, Figure 4 and Figure 5). These data suggested BML-111 played a protective role in LPS/d-GalN-induced acute liver injury by regulating the balance of inflammatory cytokines.

On the other hand, it reported that LPS/d-GalN challenge caused the overproduction of ROS, which played an important role in the development of acute liver injury [29]. Once the balance between production of ROS and the physical antioxidant defense system is broken, oxidative stress will occur and lead to liver injury. MDA is produced by lipid peroxidation mediated by free radicals and generally used as a marker of oxidative stress [30]. In addition, it has been shown that NO, mainly induced by iNOS, can cause tissue damage through reacting with superoxide anions, which may induce peroxynitrite formation [31]. In this study, we found BML-111 significantly inhibited LPS/d-GalN induced MDA and NO in rats, and also the activity of iNOS (Figure 6 and Figure 7). Meanwhile, enzymatic and non-enzymatic antioxidant mechanisms constitute important lines of defense against oxidative damage, and we also measured the hepatic activities of antioxidant enzymes such as CAT and SOD, as well as non-enzymatic antioxidant mechanisms including T-AOC and hydroxyl radical-scavenging ability. The data demonstrated that BML-111 significantly enhanced antioxidant capacity by increasing the activities of SOD and CAT, stimulating T-AOC and hydroxyl radical-scavenging ability (Figure 8). It suggested that BML-111 protected LPS/d-GalN-induced acute liver injury by improving antioxidant capacity.

In addition to inflammation and oxidative stress, apoptosis and immune responses, especially apoptosis, played vital roles in the process of live injury. Inflammation and oxidative stress are closely related to apoptosis: (1) neutrophil-mediated inflammatory injury is actually dependent on hepatic parenchymal apoptosis providing the signal for extravasation and toxicity of neutrophils [32]; (2) the combination of TNF-α and its receptoron the membrane of hepatocyte, then activating caspase-3 and inducing apoptosis [28]; and (3) oxidative stress can induce apoptosis through activating NF-κB and P53, initiating mitochondrial mediated apoptosis. Are the protective effects of BML-111 on LPS/d-GalN-induced acute liver injury related to the regulation of apoptosis? If so, what are the upstream mechanisms and downstream targets? Which are more desirable targets to prevent and treat acute liver injury? These need to be researched further.

The prevention of the transition from acute to chronic inflammation is important for protecting against excessive injury, and promoting the restoration of tissue structure and function. Currently used drugs for the treatment of inflammation diseases, such as non-steroidal anti-inflammatory drugs (NSAIDs), are based on interrupting the synthesis and actions of pro-inflammation mediators, thereby disrupting the inflammatory process. The myriad of undesirable side effects of these drugs, however, mandates a more comprehensive understanding of the endogenous pathways and cellular mechanisms that antagonize the inflammatory response and promote the natural resolution phase [33]. Since LXs possess unique dual anti-inflammatory and pro-resolving effects, we speculated that LXs might have potential as novel drugs for liver injury. Our study showed BML-111 indeed exerted a protective effect on liver injury, by improving antioxidant capacity and regulating the inflammatory cytokine balance. Thus, LXs and analogues, such as BML-111, maybe become the potential therapeutic strategy for liver injury and develop effective pharmaceutical agents. Certainly, further investigations are needed for final application.

## 4. Materials and Methods

### 4.1. Reagents

BML-111 was obtained from Cayman Chemical Company (Ann Arbor, MI, USA). LPS (*Escherichia coli*, O55:B5) and d-galactosamine (d-GalN) were purchased from Sigma Chemical Company (St. Louis, MO, USA). Rat tumor necrosis factor-α (TNF-α), interleukin-1β (IL-1β) interleukin-10 (IL-10) enzyme-linked immunosorbent assay (ELISA) kits were from BD Biosciences (Bedford, OH, USA). Transforming growth factor-β1 (TGF-β1) and cyclooxygenase-2 (COX-2) polyclonal antibodies were purchased from Anbo Biotechnology Company (San Francisco, CA, USA). Assay kits for alanine aminotransferase (ALT), aspartate aminotransferase (AST), myeloperoxidase (MPO), malondialdehyde (MDA), nitric oxide (NO), the activation of inducible nitric oxide synthase (iNOS), catalase (CAT), superoxide dismutase (SOD) and total antioxidant capacity (T-AOC) and hydroxyl radical-scavenging ability were purchased from Nanjing Jiancheng Bioengineering Institute (Nanjing, China). Caspase 3 activity assay kits were obtained from Beyotime Institute (Shanghai, China).

### 4.2. Animals and Experimental Protocols

Sprague–Dawley rats weighing 180–220 g were obtained from the Animal Center of Nanchang University (Nanchang, China) and raised in an environmentally-controlled room with a 12 h light/dark cycle and free access to food and water. Acute liver injury was induced by intraperitoneal injection of 20 μg/kg body weight of LPS and 700 mg/kg body weight of d-GalN. Forty rats were randomly divided into four groups. Rats in the control group received injections of PBS alone. Rats in the BML-111 group were injected with BML-111 (1 mg/kg) alone. Rats in the model group received LPS injections and 15 min later were injected with d-GalN. Rats in prevention group received BML-111 (1 mg/kg) by intraperitoneal injection 30 min prior to injection with LPS and d-GalN as in the model group. The study was performed according to the institution’s guiding principles for the care and use of laboratory animals.

### 4.3. Preparation of Serum and Tissue Samples

For the experiment, 1.5, 6 or 8 h after administration of LPS/d-GalN, rats were anesthetized and serum were collected. The livers were dissected, washed, homogenized with cold saline and centrifuged, before removal of the supernatant. Serum and liver homogenate supernatants were stored at −80 °C.

### 4.4. Histopathological Evaluation

Eight hours after administration of LPS/d-GalN, liver tissues were collected and then fixed in 10% neutral buffered formalin and embedded in paraffin. In addition, 4 μm slices were prepared and stained with haematoxylin and eosin, according to standard procedures.

### 4.5. Neutrophil Infiltration Assay (MPO Assay)

As an index of neutrophil infiltration, tissue-associated MPO activity was determined. Briefly, 8 h after administration of LPS/d-GalN, liver tissues were homogenized in a phosphate buffer (20 mmol/L, pH 7.4). After centrifugation at 30,000× *g* for 30 min, the pellet was resuspended in another potassium phosphate buffer (50 mmol/L, pH 6.0) with 0.5% hexadecyltrimethyl ammonium bromide. Samples were centrifuged at 20,000× *g* for 15 min at 4 °C, and supernatants were saved. MPO activity in the livers was assayed by measuring absorbance changes spectrophotometrically at 460 nm, using 0.167 mg/mL *o*-dianisidine hydrochloride and 0.0005% hydrogen peroxide. Myeloperoxidase activity per gram organ wet weight (in units/g) was calculated as (*A*_460_ × 13.5)/organ weight, where *A*_460_ is the change in absorbance of 460 nm light from 1 to 3 min after the initiation of the reaction, and 13.5 is a coefficient that was empirically determined such that one unit of MPO activity is the amount of enzyme that will reduce 1 μmol roxide/min. Results were expressed as units MPO per gram of wet tissue.

### 4.6. Liver Function Assay

Eight hours after administration of LPS/d-GalN, serum was acquired by centrifugation of blood samples 3000 rpm for 10 min. Serum AST and ALT activities were measured using the assay kits (Nanjing Jiancheng Bioengineering Institute, Nanjing, China) following the manufacturer’s instruction in a microplate reader.

### 4.7. Enzyme-Linked Immunosorbent Assay (ELISA)

Serum was acquired by centrifugation of blood samples 3000 rpm for 10 min. After preparation, the standard reagents by dilution, add 100 μL standard solution or sample into the tubes respectively, mix well, and incubate for 2 h at 37 °C. Discard the supernate, add 100 μL detection reagent A, mix well, and incubate for 1 h at 37 °C. Wash the microplates three times. Add 100 μL detection reagent B, mix well, and incubate for 0.5 h at 37 °C. Wash the microplates again five times. Add 90 μL substrate of tetramethylbenzidine (TMB), mix well, and incubate for 15–25 min at 37 °C. Add 50 μL stopping buffer and measure the absorbances at 450 nm immediately via microplate reader. The concentration was assessed by reference to the standard curve. TNF-α levels were measured at 1.5 h after the challenge of LPS/d-GalN.

### 4.8. Caspase-3 Protease Activity

Six hours after administration of LPS/d-GalN, caspase-3 protease activity in the liver tissue was measured according to the manufacturer’s instructions. In brief, homogenates of liver were centrifuged at 20,000× *g* for 15 min, the supernatant was incubated with acetyl-Asp-Glu-Val-Asp *p*-nitroanilide (Ac-DEVD-pNA), and reaction buffer for 90 min at 37 °C. Absorbance was measured at 405 nm as caspase-3 activity. 

### 4.9. Western Blotting Analysis

Liver tissues were homogenized in triple detergent lysis buffer containing 50 mmol/L Tris-HCl (pH 7.5), 150 mmol/L NaCl, 0.1% sodium dodecylsulfate (SDS), 1% Nonidet P-40, 0.5% sodium deoxycholate, 100 mg/mL phenylmethylsulfonyl fluoride (PMSF) and 1 mg/mL aprotinin. Protein concentration in the supernatant was measured in triplicate using a BCA™ Protein Assay Kit (Pierce, Rockford, IL, USA). Whole cell lysates (40 μg) were separated in 10% polyacrylamide gels and electrotransferred to nitrocellulose membranes. The nitrocellulose membranes were blocked at room temperature for 1 h in Tris-buffered saline (TBS) containing 5% milk and 0.5% Tween 20 and incubated overnight at 4 °C with primary TGF-β1 or COX-2 antibodies, prior to incubation with horseradish peroxidase-coupled secondary antibody. Detection was carried out by enhanced chemiluminescence according to the manufacturer’s protocol (Pierce, Rockford, IL, USA).

### 4.10. Evaluation of Oxidation Degree (iNOS, NO, and MDA)

Eight hours after administration of LPS/d-GalN, hepatic tissues were homogenized in cold physiological saline, centrifuged at 12,000× *g* at 4 °C for 10 min, and then supernatants were collected. The activity of iNOS, the concentration of NO, and the amount of MDA were tested via corresponding assay kits according to the manufacturer's instructions (Jiancheng Institute of Biotechnology, Nanjing, China).

### 4.11. Measurement of Malondialdehyde (MDA)

The level of MDA, an indicator of lipid peroxidation, was determined using an assay kit (Nanjing Jiancheng Bioengineering Institute, Nanjing, China) following the manufacturer’s instruction. The principle of the method is the spectrophotometric measurement of the color generated by the reaction of thiobarbituric acid with MDA. For this purpose, liver tissues were weighed and homogenized (1:10, *w*/*v*) in 0.1 mol/L phosphate buffer (pH 7.4) in an ice bath. The homogenates were centrifuged at 3000× *g* for 20 min at 4 °C. MDA levels in the supernatants were measured strictly following the recommendations of the manufacturer. The protein content of the supernatant was determined by BCA Protein Assay Kit (Pierce, Rockford, IL, USA) and the concentration of MDA was expressed as nanomoles per milligram of protein.

### 4.12. Statistical Analysis

All statistical analyses were performed using SPSS 19.0 (IBM, Armonk, NY, USA). The data were expressed as mean ± SD and subjected to statistical analysis one-way ANOVA. Differences were taken statistically significant when *p* < 0.05.

## 5. Conclusions

BML-111 played a protective role in acute liver injury induced by LPS and d-GalN in rats, through improving antioxidant capacity and regulating the balance of inflammatory cytokines.

## References

[1] Brink C., Dahlén S.E., Drazen J., Evans J.F., Hay D.W., Nicosia S., Serhan C.N., Shimizu T., Yokomizo T. (2003). International Union of Pharmacology XXXVII. Nomenclature for leukotriene and lipoxin receptors. Pharmacol. Rev..

[2] Fierce I.M., Serhan C.N. (2001). Mechanisms in anti-inflammation and resolution: The role of lipoxins and aspirin-triggered lipoxins. Braz. J. Med. Biol. Res..

[3] Serhan C.N. (2005). Lipoxins and aspirin-triggered 15-epi-lipoxins are the first lipid mediators of endogenous anti-inflammation and resolution. Prostaglandins Leukot. Essent. Fat. Acids.

[4] Chiang N., Takano T., Arita M., Watanabe S., Serhan C.N. (2003). A novel rat lipoxin A4 receptor that is conserved in structure and function. Br. J. Pharmacol..

[5] Serhan C.N., Maddox J.F., Petasis N.A., Akritopoulou-Zanze I., Papayianni A., Brady H.R., Colgan S.P., Madara J.L. (1995). Design of lipoxin A4 stable analogs that block transmigration and adhesion of human neutrophils. Biochemistry.

[6] Lee T.H., Lympany P., Crea A.E., Spur B.W. (1991). Inhibition of leukotriene B4-induced neutrophil migration by lipoxin A4: Structure-function relationships. Biochem. Biophys. Res. Commun..

[7] Gong J., Guo S., Li H.B., Yuan S.Y., Shang Y., Yao S.L. (2012). BML-111, a lipoxin receptor agonist, protects haemorrhagic shock-induced acute lung injury in rats. Resuscitation.

[8] Wang Y.Z., Zhang Y.C., Cheng J.S., Ni Q., Li P.J., Wang S.W., Han W., Zhang Y.L. (2014). BML-111, a lipoxin receptor agonist, ameliorates “two-hit”-induced acute pancreatitis-associated lung injury in mice by the upregulation of heme oxygenase-1. Artif. Cells Nanomed. Biotechnol..

[9] Li H.B., Wang J.Z., Gong J., Wu Z.Y., Guo S., Li B., Liu M., Ji Y.D., Tang M., Yuan S.Y. (2013). BML-111 attenuates hemorrhagic shock-induced acute lung injury through inhibiting activation of mitogen-activated protein kinase pathway in rats. J. Surg. Res..

[10] Gong J., Li H.B., Guo S., Shang Y., Yao S.L. (2012). Lipoxin receptor agonist, may be a potential treatment for hemorrhagic shock-induced acute lung injury. Med. Hypotheses.

[11] Tang M., Chen L., Li B., Wang Y., Li S., Wen A., Yao S., Shang Y. (2016). BML-111 attenuates acute lung injury in endotoxemic mice. J. Surg. Res..

[12] Zhang L., Zhang X., Wu P., Li H., Jin S., Zhou X., Li Y., Ye D., Chen B., Wan J. (2008). BML-111, a lipoxin receptor agonist, modulates the immune response and reduces the severity of collagen-induced arthritis. Inflamm. Res..

[13] Liu H., Liu Z., Zhao S., Sun C., Yang M. (2015). Effect of BML-111 on the intestinal mucosal barrier in sepsis and its mechanism of action. Mol. Med. Rep..

[14] Wu S.H., Chen X.Q., Lü J., Wang M.J. (2016). BML-111 Attenuates renal ischemia/reperfusion injury via peroxisome proliferator-activated receptor-α-regulated heme oxygenase-1. Inflammation.

[15] Hawkins K.E., DeMars K.M., Singh J., Yang C., Cho H.S., Frankowski J.C., Doré S., Candelario-Jalil E. (2014). Neurovascular protection by post-ischemic intravenous injections of the lipoxin A4 receptor agonist, BML-111, in a rat model of ischemic stroke. J. Neurochem..

[16] Zhou X.Y., Yu Z.J., Yan D., Wang H.M., Huang Y.H., Sha J., Xu F.Y., Cai Z.Y., Min W.P. (2013). BML-111, A lipoxin receptor agonist, protected carbon tetrachloride-induced hepatic fibrosis in rats. Inflammation.

[17] Zhou X.Y., Li Y.S., Wu P., Wang H.M., Cai Z.Y., Xu F.Y., Ye D.Y. (2009). Lipoxin A4 inhibited hepatocyte growth-induced invasion of human hepatoma cells. Hepatol. Res..

[18] Hao H., Liu M., Wu P., Cai L., Tang K., Yi P., Li Y., Chen Y., Ye D. (2011). Lipoxin A4 and its analog suppress hepatocellular carcinoma via remodeling tumor microenvironment. Cancer Lett..

[19] Chen Y., Hao H., He S., Cai L., Li Y., Hu S., Ye D., Hoidal J., Wu P., Chen X. (2010). Lipoxin A4 and its analogue suppress the tumor growth of transplanted H22 in mice: The role of anti-angiogenesis. Mol. Cancer Ther..

[20] Silverstein R. (2004). D-Galactosamine lethality model: Scope and limitations. J. Endotoxin Res..

[21] Wilhelm E.A., Jesse C.R., Roman S.S., Nogueira C.W., Savegnago L. (2009). Hepatoprotective effect of 3-alkynyl selenophene on acute liver injury induced by d-galactosamine and lipopolysaccharide. Exp. Mol. Pathol..

[22] Zhang L., Wan J., Li H., Wu P., Jin S., Zhou X., Yuan P., Xiong W., Li Y., Ye D. (2007). Protective effects of BML-111, a lipoxin A4 receptor agonist, on carbon tetrachloride-induced liver injury in mice. Hepatol. Res..

[23] El-Agamy D.S., Makled M.N., Gamil N.M. (2014). Protective effects of BML-111 against acetaminophen-induced acute liver injury in mice. J. Physiol. Biochem..

[24] Ning Q., Brown D., Parodo J., Cattral M., Gorczynski R., Cole E., Fung L., Ding J.W., Liu M.F., Rotstein O. (1998). Ribavirin inhibits viral induced macrophage production of TNF, IL-1, the procoagulant fgl2 prothrombinase and preserves Th1 cytokine production but inhibits Th2 cytokine response. J. Immunol..

[25] White B., Schmidt M., Murphy C., Livingstone W., O’Toole D., Lawler M., O’Neill L., Kelleher D., Schwarz H.P., Smith O.P. (2000). Activated protein C inhibits lipo-polysaccharide-induced nuclear translocation of nuclear factor κB (NF-κB) andtumour necrosis factor α (TNF-α) production in the THP-1 monocytic cell line. Br. J. Haematol..

[26] Hishinuma I., Nagakawa J., Hirota K., Miyamoto K., Tsukidate K., Yamanaka T., Katayama K., Yamatsu I. (1990). Involvement of tumor necrosis factor-α in development of hepatic injury in galactosamine-sensitized mice. Hepatology.

[27] Tiegs G., Wolter M., Wendel A. (1989). Tumor necrosis factor is a terminal mediator in galactosamine/endotoxin-induced hepatitis in mice. Biochem. Pharmacol..

[28] Jaeschke H., Fisher M.A., Lawson J.A., Simmons C.A., Farhood A., Jones D.A. (1998). Activation of caspase 3 (CPP32)-like proteases is essential for TNF-α-induced hepatic parenchymal cell apoptosis and neutrophil-mediated necrosis in a murine endotoxin shock model. J. Immunol..

[29] Lu Y., Bao X., Sun T., Xu J., Zheng W., Shen P. (2012). Triptolide attenuate the oxidative stress induced by LPS/d-GalN in mice. J. Cell. Biochem..

[30] Gil L., Siems W., Mazurek B., Gross J., Schroeder P., Voss P., Grune T. (2006). Age-associated analysis of oxidative stress parameters in human plasma and erythrocytes. Free Radic. Res..

[31] Habib S., Ali A. (2011). Biochemistry of nitric oxide. Indian J. Clin. Biochem..

[32] Zhang L., Li H.Z., Gong X., Luo F.L., Wang B., Hu N., Wang C.D., Zhang Z., Wan J.Y. (2010). Protective effects of Asiaticoside on acute liver injury induced by lipopolysaccharide/d-galactosamine in mice. Phytomedicine.

[33] Chiang N., Serhan C.N., Dahlén S.E., Drazen J.M., Hay D.W., Rovati G.E., Shimizu T., Yokomizo T., Brink C. (2006). The lipoxin receptor ALX: Potent ligand-specific and stereoselective actions in vivo. Pharmacol. Rev..

